# Fetal Nerve Cell Transplantation in Early Post-Resuscitation Period in Rats

**DOI:** 10.5195/cajgh.2014.178

**Published:** 2014-12-12

**Authors:** Damira Tazhibayeva, Farkhad Olzhayev, Natalya Kadbudalieva, Zhaina Aitbayeva, Lina Zaripova

**Affiliations:** 1 Department of Pathological Physiology, Astana Medical University, Astana, Kazakhstan; 2 Center for Life Sciences, Nazarbayev University, Astana, Kazakhstan

**Keywords:** fetal nerve cell transplantation, survival, resuscitation

## Abstract

**Introduction:**

Fetal cell transplantation is a promising biomedical approach for disease treatment; however, the use of fetal cell therapy is still experimental. This research was deemed a necessity to provide evidence-based research for the application of cell transplantation as a treatment method. The aim of this study was to evaluate the effect of fetal nerve cell transplantation in rat survivors (and non-survivors) after clinical death by mechanical asphyxia.

**Methods:**

68 white laboratory rats were divided into two groups of identical age and sex: a control group of 12-month adult male rats (n = 26) and an experimental group (n = 42). Rats were fixed under ether anesthesia. We then blocked the oral and nasal regions with cotton wool soaked in saline solution. A four-minute clinical death though acute mechanical asphyxia was simulated by applying the method of N. Shim. After the 4-minute clinical death, we resuscitated the rats using external cardiac massage and artifical respiration. Suspension of the fetal nerve cells was injected intraperitoneally at 1mm^3^ per 25g at the time of cardiac activity restoration. Lactate dehydrogenase (LDH) and creatine phosphokinase (CPK) levels were examined in the homogenate cerebral cortex of reanimated animals. We recorded the survival rate during the post-resuscitation period and analyzed the integrative brain functions using anxiety-phobic status and latent inhibition.

**Results:**

After fetal nerve cell transplantation, the enzymatic reactions in the experimental group became normal with a significant decrease in LDH and an increase in CPK levels compared to the control group. In the control group, 10 rats died and 16 lived (62% survival rate), while 7 rats died and 35 lived (83% survival rate) in the experimental group during the first 7 days. Rats that did not receive the treatment tended to die sooner than those in the experimental group. As a result of transplantation, the anxiety level in the experimental group was less than in the control group. Moreover, cell therapy improved the reflexes in the experimental animals.

**Conclusions:**

The study revealed the positive neuroprotective effect of the fetal nerve cells on the recovery in the early post-resuscitation period. This was confirmed by the normalization of enzymatic reactions, improvement reflective activity, and increase in the survival rate of the resuscitated animals in the group treated with fetal nerve cell transplantation. These findings warrant future research on the mechanisms associated with reflex improvement.

